# Mixed seeding with sheep manure amendment alters soil nematode network complexity in a sandy grassland

**DOI:** 10.3389/fpls.2026.1912216

**Published:** 2026-09-29

**Authors:** Maidinuer Abulaizi, Jianqin Zhou, Mo Chen, Tianle Kou, Yuanbin Jia, Yunpeng Hu, Yuxin Tian, Yang Hu, Hongtao Jia

**Affiliations:** 1 College of Grassland Science, Xinjiang Agricultural University, Urumqi, China; 2 College of Resources and Environment, Xinjiang Agricultural University, Urumqi, China; 3 College of Resources and Environment, Xinjiang Key Laboratory of Soil and Plant Ecological Processes, Urumqi, China; 4 College of Life Science, Xinjiang Agricultural University, Urumqi, China

**Keywords:** alpine grassland desertification, mixed seeding, network complexity, sheep manure amendment, soil nematodes

## Abstract

**Introduction:**

Soil nematodes are key bioindicators of terrestrial ecosystem functions, yet how restoration practices affect their community and interactions remains unclear.

**Methods:**

A one-year field experiment in an alpine grassland tested three treatments: bare sandy soil, mixed seeding of *Avena sativa L*. and *Festuca arundinacea*, and mixed seeding with sheep manure amendment at 1.75 kg m^-^². Notably, the experimental design did not include a sheep-manure-only treatment; therefore, the specific contribution of manure independent of vegetation reconstruction cannot be disentangled.

**Result:**

High-throughput sequencing and co-occurrence network analysis revealed that mixed seeding with manure significantly enhanced plant biomass, soil organic carbon, electrical conductivity, and nematode richness (Chao1 index) by 26.36-31.58% (*p* < 0.05). Mixed seeding alone increased the relative abundance of predator/omnivore nematodes by 17.46%, whereas mixed seeding with sheep manure shifted the community toward opportunistic bacterivores such as *Acrobeloides* and reduced predators/omnivores by 15.74% (*p* < 0.05). Available nitrogen showed the strongest marginal association with community composition (Monte Carlo test, *p* = 0.065). Network analysis indicated that mixed seeding promoted the key genus *Aporcelaimellus*, while adding manure shifted the network architecture toward bacterivores dominance.

**Discussion:**

Both the mixed seeding treatment and the mixed seeding combined with manure treatment enhanced network complexity but increased competition over cooperation, reflecting altered interaction patterns. Whether this structural complexity translates into functional stability requires long-term monitoring. In conclusion, mixed seeding alone fosters a more structured soil food web with higher trophic-level representation, whereas the combination of mixed seeding with organic amendment—though boosting productivity and richness—induces salinization risks (elevated EC) and functional simplification toward bacterivore-dominated channels. The absence of a manure-only control limits our ability to attribute these effects solely to organic fertilization; future studies should include this treatment to isolate independent and interactive effects.

## 1 Introduction

As one of the most widely distributed grassland ecosystems globally, alpine grasslands underpin biogeochemical cycles and climate regulation (Zhang et al., 2025). However, influenced by human activities and climate change, these ecosystems are facing severe degradation, with the rate of deterioration currently outpacing global restoration efforts (Zhao W. et al., 2024; Wu et al., 2024). A case in point is the Bayinbuluk Alpine Grassland in the arid regions of Central Asia, where climate change and anthropogenic pressures have caused significant degradation. By 2021, degraded areas (including desertification and salinization) had expanded to cover one-third of the total grassland area (Hu et al., 2024; Abulaizi et al., 2025). Desertification represents a key threat, severely disrupting ecosystem structure and leading to soil nutrient loss, declining productivity, and reduced vegetation cover (D’Odorico et al., 2013; Liu et al., 2021). Existing studies indicate that degradation can trigger a sharp decline in vegetation cover to approximately 15% and significantly alter the composition of soil bacterial communities (Abulaizi et al., 2023). In terms of belowground ecological processes, previous research has confirmed that grassland degradation reduces soil nematode diversity and simplifies their food web structure (Hu et al., 2024; 2025a). These findings highlight the close coupling between aboveground vegetation and belowground biological networks.

To address grassland degradation, two primary restoration approaches have been developed: natural restoration (such as grazing exclusion and rotational grazing) and active intervention (e.g., sowing, fertilization, establishment of artificial grasslands, and sand barriers) (Li et al., 2023; Zhao W. et al., 2024; Wu et al., 2024; Fan et al., 2025; Wang et al., 2020). Among these, restoring community structure through the establishment of high-coverage vegetation has been widely adopted (Zhao W. et al., 2024). Studies show that mixed plant communities can enhance vegetation coverage, plant diversity, and biomass more effectively than monocultures (Kumar et al., 2017; Craven et al., 2016; Yan et al., 2022). Furthermore, the combined application of amendments (particularly organic fertilizer) can significantly increase aboveground biomass, improve soil nutrient content and water-holding capacity, and thus accelerate restoration (Hu et al., 2025b; Han et al., 2021). Organic fertilizer inputs can also enhance plant nutrient use efficiency by stimulating microbial activity and promoting nutrient cycling, which in turn may influence the structure and diversity of the soil food web — a key aspect investigated in this study (Fan et al., 2025). Restored ecosystems have also been shown to exhibit greater resistance and resilience (Blois et al., 2013; Li et al., 2020). However, current restoration measures generally face challenges such as long recovery period, insufficient system stability and environmental constraints (Wang et al., 2018). Additionally, artificially constructed communities often lack species diversity, which may lead to simplified ecosystem functions and services in restored areas (Singh et al., 2012; Dong et al., 2020). Therefore, optimizing planting and soil management to restore plant and soil functions has become a key research focus, particularly for combating desertification as extreme stage of degradation (Li et al., 2023).

Soil biology serves as a crucial indicator for understanding biogeochemical cycles and evaluating ecosystem restoration (Falkowski et al., 2008; Banning et al., 2011). As a core component of the soil food web, soil nematodes play key roles in organic matter decomposition, nutrient transformation, and energy transfer (Zhao et al., 2021; Fan et al., 2025). Due to their high sensitivity to environmental disturbances, nematodes are considered ideal indicators of soil ecosystem changes (Zhang et al., 2019; Ikoyi et al., 2021). Consequently, the influence of organic amendments on nematode communities has attracted increasing attention (Zhang et al., 2022). Restoration measures can directly or indirectly affect nematode community composition by altering plant communities and soil environments, including through changes in food resources (Zhang et al., 2019; Zhao et al., 2021; Zhi et al., 2008). For instance, organic amendments may reduce nematode richness and diversity (Sarathchandra et al., 2001; Liang et al., 2009), and such changes in nematode communities can exert top-down effects on other trophic levels, such as microbial communities (Chen et al., 2014b; Zhang et al., 2019). In particular, predatory and omnivorous nematodes play a crucial role in regulating lower trophic levels, and their presence is widely recognized as an indicator of food web complexity and ecosystem stability. Although various ecological restoration measures have been implemented, current research largely focuses on the responses of vegetation communities and soil physicochemical properties, while the response mechanisms of belowground soil biota (particularly key indicator groups like soil nematodes) remain insufficiently studied. This knowledge gap is very important, because soil nematodes play vital roles in regulating nutrient cycle, decomposition pathway and soil food web dynamics, and their neglect limits our comprehensive evaluation of restoration results.

Based on this, this study selected mixed seeding of *Avena sativa* and *Festuca arundinacea* as a vegetation reconstruction measure, and examined the additional effect of combining this mixed seeding with sheep manure amendment, for the following reasons: mixed seeding is widely used to enhance vegetation diversity and productivity, while the addition of sheep manure to mixed seeding represents a distinct intervention pathway that may accelerate restoration through direct nutrient input. It is important to note that the experimental design compares mixed seeding alone versus mixed seeding with manure, rather than testing organic fertilization as an independent factor. The specific aims were (1) to compare the effects of mixed seeding alone versus mixed seeding combined with sheep manure on the diversity, composition, and structure of soil nematode communities in a desertified alpine grassland; and (2) to identify the key environmental factors driving these changes by simultaneously measuring soil physicochemical properties (e.g., pH, organic carbon, total nitrogen, phosphorus) and linking them to nematode community metrics through multivariate statistical analyses. Based on the above, we propose the following hypotheses: (1) mixed seeding, especially when combined with sheep manure amendment, will significantly alter soil physicochemical properties (for example, increasing soil organic matter and nutrient availability) and aboveground vegetation characteristics. (2) These alterations will, in turn, enhance soil nematode diversity and shift community composition toward a more structured, resource-rich state, because improved vegetation and soil conditions create a favorable microhabitat (here defined as the localized soil environment influencing nematode survival and activity, including pH, organic matter distribution, and pore space).

## 2 Materials and methods

### 2.1 Description of the study area

This study was conducted in a desertified area within the Bayinbuluk National Swan Nature Reserve, Xinjiang, China. The study area was located between 84°28′33.6″-84°28′55.2″ E and 42°42′36.0″-42°42′43.2″ N, at an elevation ranging from 2300 to 3042 m. The region experiences a typical alpine climate, with an average annual temperature of -6.6 °C and an average annual precipitation of 273 mm (Hu et al., 2024). The soil is predominantly sandy grassland soil, and the vegetation community is dominated by *Leymus secalinus*. The growing season generally extends from mid-May to early September. The specific location of the study site is presented in **Figure 1**.

**Figure 1:**
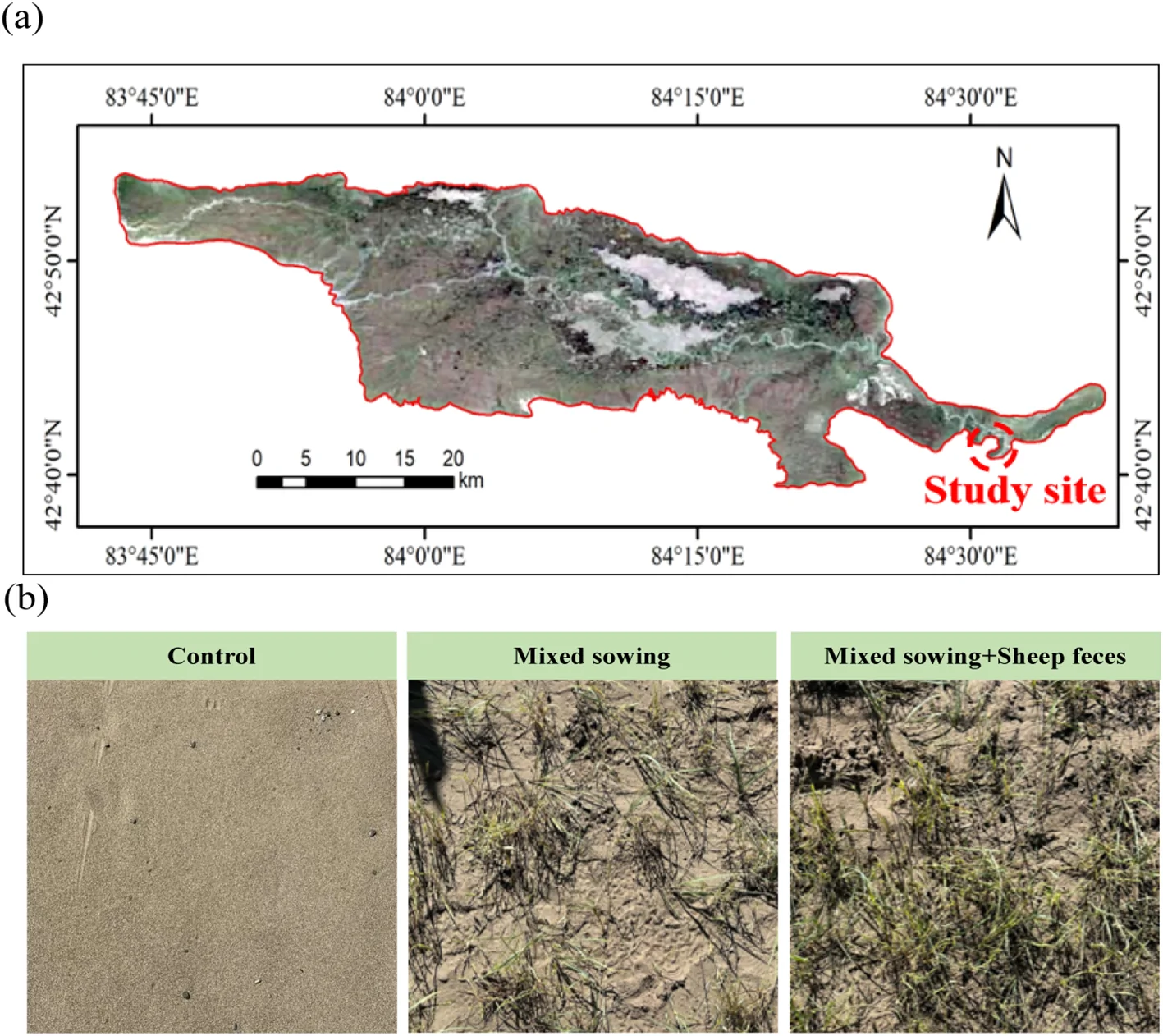
Location of study area **(a)** and effect diagram of restoration test **(b)**.

### 2.2 Experimental design

A one-year field experiment was conducted within the Bayinbuluk National Nature Reserve (Xinjiang, China) to evaluate the effects of restoration measures on desertified alpine grassland. Three spatially independent sites (>1 km apart, each approximately 600 m²) were selected on relatively flat terrain (**Figure 1**). Within each site, three restoration treatments were assigned to 3 m × 3 m sub-plots: (1) CK: bare sandy soil (Vegetation coverage was less than 15%, no fertilizer); (2) T1: mixed seeding of *Avena sativa* L. and *Festuca arundinacea*, each at 15 g m^-^²; (3) T2: same mixed seeding as T1 plus sheep manure at 1.75 kg m^-^² (≈17.5 t ha^-^¹). This manure rate was selected based on local farmers’ practice in the region (Hu et al., 2024). Each treatment was replicated five times per site, giving a total of 45 experimental plots (3 sites × 3 treatments × 5 replicates).

The experiment commenced in May 2024. Initial soil physicochemical properties showed no significant differences among the three restoration measures. The ranges were as follows: soil organic matter, 8.69-10.69 g kg^-1^; available nitrogen, 31.27-38.53 mg kg^-1^; available phosphorus, 0.85-0.92 mg kg^-1^; available potassium, 49.56-55.33 mg kg^-1^; pH, 8.50-8.57; and electrical conductivity, 340.48-361.49 μS cm^-1^. The sheep manure composition ranges were: organic carbon, 497.87-547.82 g kg^-1^; total nitrogen, 45.04-57.08 g kg^-1^; and total phosphorus, 2.31-2.87 g kg^-1^.

### 2.3 Sample collection and index determination

The grassland restoration experiment was conducted across three enclosed plots. Within each plot, three restoration measures were implemented, with five replicates per measure, resulting in a total of 45 experimental sub-plots (3 plots × 3 measures × 5 replicates). Each sub-plot measured 3 × 3 m.

For vegetation monitoring, three 1 × 1 m quadrats were established within each sub-plot, spaced at least 0.5 m apart, to measure plant height and coverage (Hu et al., 2024). Aboveground biomass was harvested by clipping all plants at ground level within a designated area. Simultaneously, a soil core (0–10 cm depth) was collected from each quadrat using a 5 cm diameter soil auger for the determination of root biomass (Hu et al., 2024) (**Figure 1**).

Soil samples were collected in the quadrat (3 m×3 m) used for plant sampling. In each quadrat, a soil drill with a diameter of 7 cm and a length of 20 cm is used to collect 20 soil cores from the 0–10 cm topsoil layer along an “S” shape and mix them into a composite sample. Subsequently, the composite sample was screened by a 2 mm sterile screen to remove gravel and roots, and was evenly mixed and divided into three parts: one part was air-dried at room temperature for soil physical and chemical properties analysis; One copy is stored at -80 °C for subsequent extraction of nematode DNA; The other copy is stored at 4 °C for other analyses. After the collected plant samples are brought back to the laboratory, they are dried to a constant weight (at least 48 hours) in an oven at 65 °C, and the dry weights of the aboveground and underground parts are respectively weighed to calculate the aboveground biomass and underground biomass.

Soil physicochemical properties, including soil organic carbon (determined by the sulfuric acid oxidation method), alkaline hydrolysis nitrogen (by the alkali diffusion method), available phosphorus (by sodium bicarbonate extraction-molybdenum antimony colorimetry), available potassium (by flame photometry combined with sodium hydroxide fusion-flame photometry), pH, and electrical conductivity (measured with a pH meter and a conductivity meter, respectively), were analyzed following the standard methods described in Soil and agro-chemical analysis (Bao et al., 2015).

### 2.4 DNA extraction, sequencing, and bioinformatics analysis

Total DNA of the soil nematode community was extracted using the DNeasy PowerSoil Pro Kit (QIAGEN, Germany). The V4-V5 region of the 18S rRNA gene was amplified via PCR using nematode-specific primers NF1 (5′-GGTGGTGCATGGCCGTTCTTAGTT-3′) and 18Sr2b (5′-TACAAAGGGCAGGGACGTAAT-3′). The amplification products were verified by 1.8% agarose gel electrophoresis and then purified using the Agencourt AMPure XP purification kit. The purified products were subjected to a second round of PCR amplification with indexed primers to construct the sequencing library. The constructed libraries were quantified and assessed for fragment size distribution using a Qubit 4.0 fluorometer and an Agilent 4200 Bioanalyzer. Finally, paired-end 250 bp sequencing was performed on the Illumina NovaSeq 6000 platform (Illumina, USA).

Raw sequencing data were processed for quality control: primers and low-quality sequences were first removed using Trimmomatic (v0.33) and Cutadapt (v1.9.1); subsequently, denoising, sequence merging, and chimera removal were performed via the DADA2 (v2020.6) pipeline in QIIME2, generating amplicon sequence variants (ASV) of the 18S rRNA gene. ASVs were taxonomically classified using a naïve Bayes classifier with reference databases specialized for eukaryotes or nematodes (e.g., Silva 138 SSU Ref NR99 or the PR2 database). Based on the classification results, ASVs assigned to the phylum Nematoda were retained for subsequent community statistical analyses.

Taxonomic assignment and quality control. ASVs were taxonomically classified using a naïve Bayes classifier trained on the SILVA 138 SSU Ref NR99 database (Quast et al., 2013) and the PR2 database v.4.14.0 (Guillou et al., 2013), implemented in QIIME 2 (v2020.6). Classification was performed with a confidence threshold of 0.8. Only ASVs assigned to the phylum Nematoda at ≥ 80% confidence were retained; non-nematode and non-target reads were filtered out at this step. ASVs that failed genus-level classification were retained at the highest resolved taxonomic rank (family or order) and labeled as unclassified.

Functional group assignment. Nematodes were assigned to trophic groups (bacterivores, fungivores, plant parasites, predators/omnivores) based on the classification system of Yeates et al. (1993) and Bongers and Bongers (1998), and Ferris et al. (2001), supplemented by genus-level feeding habit references from the Nemaplex database (https://nemaplex.ucdavis.edu).

### 2.5 Data analysis

To test the effects of restoration measures on plant, soil, and nematode α-diversity indicators, linear mixed-effects models (LMMs) were fitted using the lmer function in R (package lme4). In these models, treatment (CK, T1, T2) was included as a fixed factor, while site (three spatially independent blocks) was treated as a random intercept to account for the nested spatial structure. Denominator degrees of freedom were estimated using the Satterthwaite approximation (lmerTest package). *Post-hoc* pairwise comparisons were performed using estimated marginal means (emmeans) with Tukey’s adjustment for multiple comparisons, replacing the original LSD tests. The complete LMM outputs are provided in **Supplementary Table 1**. The α-diversity indices (e.g., Chao1 and Shannon) were calculated in QIIME2 (v2019.4) and visualized using the ggplot2 package in R. Based on the Bray–Curtis distance matrix of nematode community composition, principal coordinate analysis (PCoA) was conducted using the vegan package in R. Differences in community composition among treatments were primarily tested using permutational multivariate analysis of variance (PERMANOVA; adonis2, 999 permutations). Analysis of similarities (ANOSIM; anosim, 999 permutations) was additionally performed as a complementary rank-based test to verify the robustness of the PERMANOVA result. Homogeneity of multivariate dispersion was assessed using the betadisper function.

To identify nematode taxa with significantly differential abundance among treatment groups (i.e., biomarkers), linear discriminant analysis effect size (LEfSe) analysis was applied. To explore the driving effect of environmental factors on nematode community structure, redundancy analysis (RDA) coupled with Monte Carlo permutation tests (9999 permutations) was performed. To reveal the internal interaction relationships within nematode communities under different treatments, co-occurrence networks were constructed at the ASV level. Specifically, network topology parameters (such as average degree, clustering coefficient, and modularity) were calculated using the vegan and igraph packages in R, and network visualization was generated using Gephi software.

Co-occurrence networks were constructed at the ASV level using Spearman rank correlations based on relative abundance data. To address the compositional nature of sequencing data, centered log-ratio (CLR) transformation was applied prior to correlation analysis (Aitchison, 1986). Only ASV present in ≥ 50% of samples within each treatment were retained to reduce spurious correlations driven by rare taxa. Correlation coefficients with |ρ| > 0.6 and adjusted *p*-values < 0.05 (Benjamini-Hochberg FDR correction) were considered significant. Networks were treated as undirected because edges were derived from Spearman correlations. Reciprocal duplicate records were collapsed before analysis. Average degree was calculated as 2E/N. Diameter and average path length were calculated within the largest connected component. For weighted analyses, correlation strength was converted to distance as 1 − |ρ|.

## 3 Results

### 3.1 Changes in environmental factors

Both mixed seeding (T1) and mixed seeding combined with sheep manure (T2) significantly increased aboveground and belowground biomass compared to the bare sandy soil control (CK; LMM, *p* < 0.001 for both; **Supplementary Table 1**). Aboveground biomass was 70.78%-97.12% higher under T1 and 63.06%-88.21% higher under T2 relative to CK, with T2 showing marginally greater biomass than T1 in some site replicates (**Figure 2**). Belowground biomass followed a similar pattern, confirming that vegetation reconstruction effectively restored plant productivity in this desertified alpine grassland.

**Figure 2:**
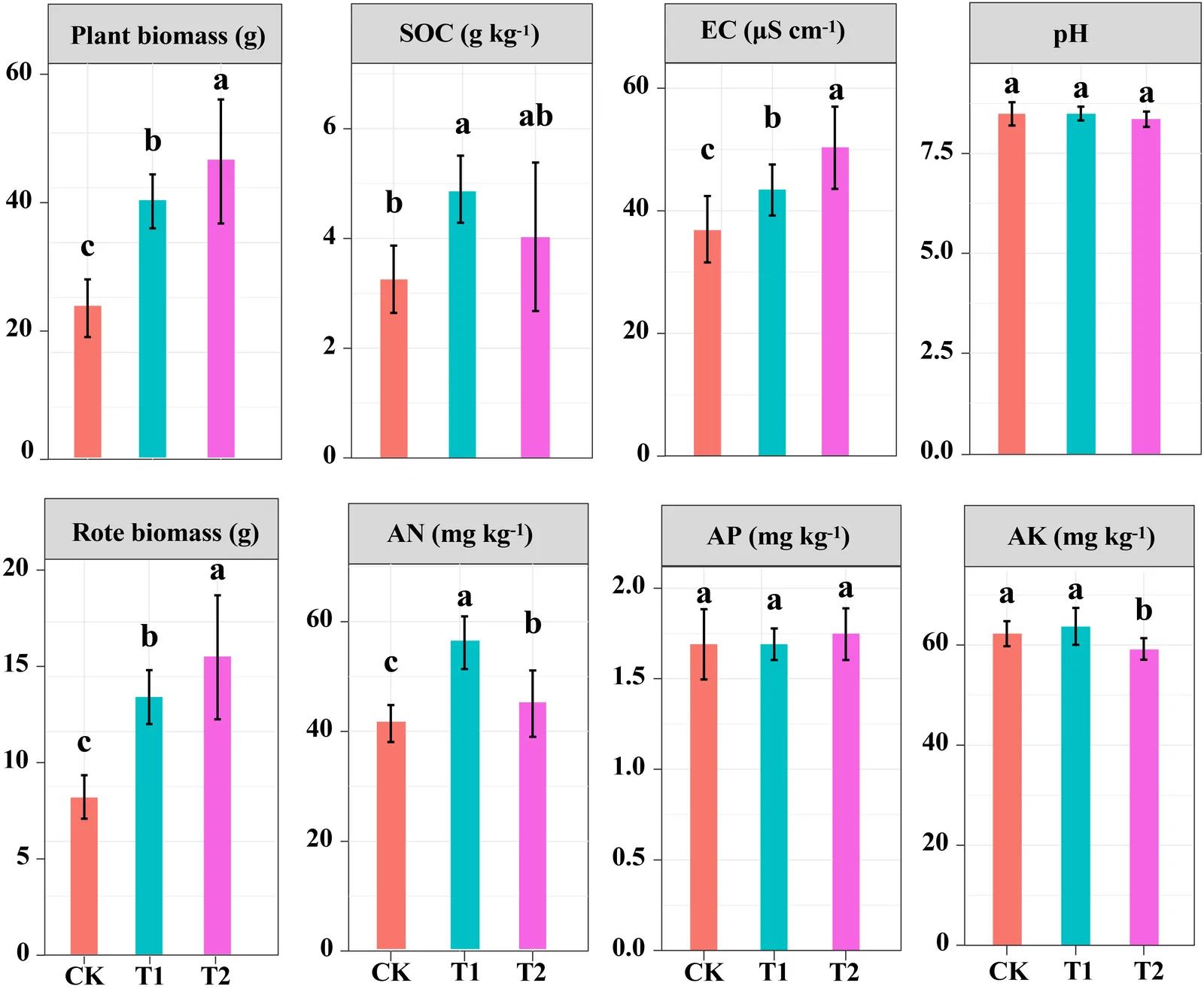
Effects of treatments on plant biomass and soil properties. Different lowercase letters indicate significant differences at *P* < 0.05 based on linear mixed-effects models followed by Tukey’s HSD post-hoc tests (see **Supplementary Table 1** for detailed statistics).

Soil organic carbon content was significantly higher under both T1 and T2 compared to CK (LMM, *p* = 3.06 × 10^-5^; **Supplementary Table 1**), with no significant difference between the two restoration treatments (*p* = 0.214). This indicates that vegetation establishment itself, rather than the addition of sheep manure, was the primary driver of SOC accumulation during this one-year experiment. Available nitrogen (AN) showed significant differences among treatments (LMM, *p* = 7.37 × 10^-^¹^4^). Notably, AN was highest under T1 (mixed seeding alone), significantly exceeding both CK (*p* = 2.63 × 10^-^¹³) and T2 (*p* = 2.57 × 10^-^¹^0^). This unexpected pattern suggests that the direct input of sheep manure (total nitrogen: 45.04-57.08 g kg^-1^) may have triggered microbial immobilization or ammonia volatilization losses in the high-pH sandy soil (pH 8.50-8.57), thereby reducing nitrogen availability relative to the plant-driven mineralization pathway under T1. Alternatively, the rapid plant uptake under T2 may have depleted the labile nitrogen pool. This finding underscores the complexity of nitrogen dynamics in organic-amended sandy soils and cautions against assuming that manure addition linearly increases nutrient availability. Available phosphorus (AP) did not differ significantly among treatments (LMM, *p* = 0.398), reflecting the strong chemical fixation of phosphorus in alkaline soils. Available potassium (AK) was significantly lower under T2 than under both CK and T1 (*p* = 0.015 and *p* = 2.52 × 10^-4^, respectively), likely due to intensified plant uptake following the biomass surge under manure amendment. Soil pH showed no significant changes (*p* = 0.151), while electrical conductivity (EC) was significantly elevated under T2 compared to both CK and T1 (*p* < 0.001 for both), indicating a salinization risk associated with sheep manure application in this arid environment.

In summary, both restoration measures improved soil properties and increased plant biomass, but the effects differed markedly between treatments: mixed seeding alone promoted higher nitrogen availability, whereas manure addition enhanced conductivity and accelerated potassium depletion.

### 3.2 Community diversity of nematodes

Soil nematode richness (Chao1 index) differed significantly among treatments (LMM, *p* = 0.003). *Post-hoc* comparisons revealed that T2 significantly increased Chao1 richness compared to CK (Tukey-adjusted *p* = 0.002), whereas the T1 effect was marginally non-significant (*p* = 0.053). The Chao1 index was 26.36%-31.58% higher under restoration treatments relative to CK (**Figure 3a**). However, Shannon diversity did not differ significantly among treatments (LMM, *p* = 0.689; **Figure 3b**), indicating that restoration increased the number of nematode taxa but did not alter community evenness during this early restoration stage.

**Figure 3:**
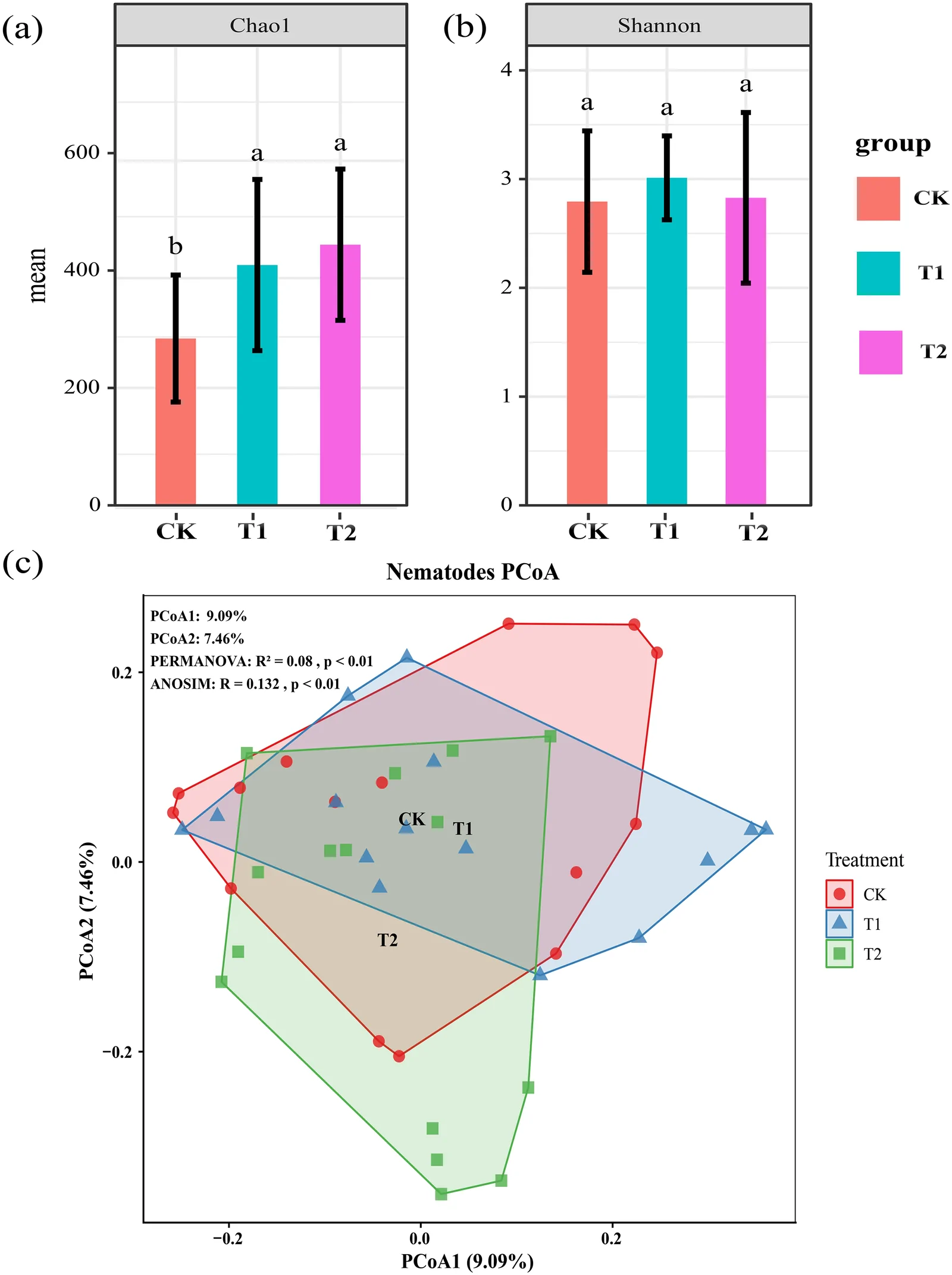
Differences in diversity of nematodes communities within among different treatment. **(a, b)** Differences in α-diversity of nematodes communities; **(c)** PCoA showing differences in soil nematodes community composition. Different lowercase letters indicate significant differences at P < 0.05 based on different treatments.

Principal coordinate analysis (PCoA) based on Bray–Curtis distance revealed significant differences in soil nematode community structure among treatments (PERMANOVA: R² = 0.08, *p* < 0.01; ANOSIM: R = 0.132, *p* < 0.01; **Figure 3c**). The separation along PCoA1 primarily reflected the contrast between CK and the two restoration treatments, while PCoA2 distinguished T1 from T2, suggesting that manure addition induced a distinct community trajectory beyond the effect of vegetation reconstruction alone.

### 3.3 Community composition and LEfSe analysis

Restoration treatments altered the trophic structure of the nematode community. Mixed seeding (T1) increased the relative abundance of predators/omnivores (e.g., *Aporcelaimellus*, with relative abundances of 49.52%, 58.02%, and 41.70% in CK, T1, and T2, respectively). The relative abundance of bacterivores slightly decreased under T1 but increased rapidly under T2. Plant parasites increased under T2 compared to CK and T1 (**Figure 4a**).

**Figure 4:**
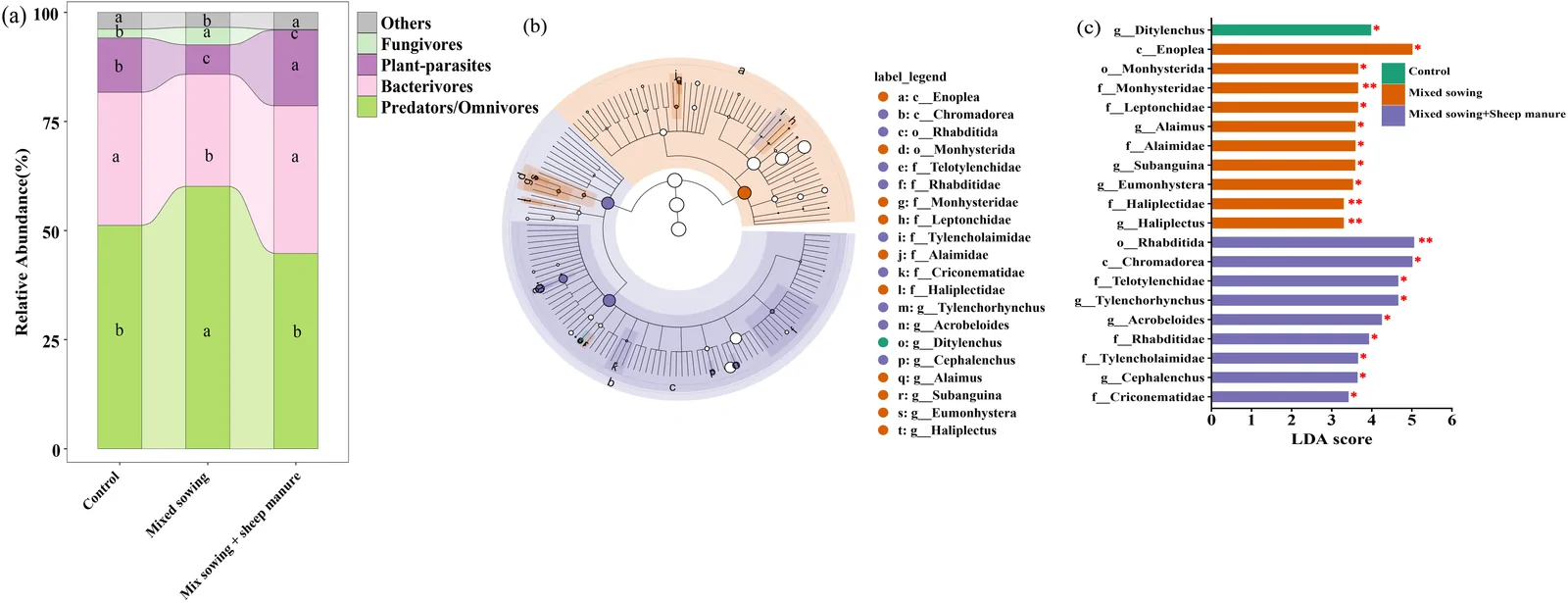
Nematodes community composition and biomarker analysis. **(a)** Changes in the relative abundance of the top 50 nematode genera, grouped by trophic groups. **(b, c)** Results of the linear discriminant analysis effect size (LEfSe) analysis identifying differential taxa among treatments. Different lowercase letters indicate significant differences at P < 0.05 based on different treatments.

LEfSe analysis identified 20 significantly different nematode taxa at LDA score ≥ 2. The CK, T1, and T2 treatments exhibited 1, 10, and 9 biomarkers with the highest relative abundances, respectively. Only the genus *Ditylenchus* was identified as a biomarker in CK. In T1, the class Enoplea showed the highest relative abundance, whereas in T2, the order Rhabditida had the highest relative abundance (**Figure 4b**), consistent with the proliferation of fast-reproducing bacterivorous nematodes under nutrient-enriched conditions.

### 3.4 Nematode community structure

Network analysis revealed that the genera *Aporcelaimellus* and *Acrobeles* were the dominant taxa across all treatments, accounting for 53.60%, 54.40%, and 57.60% (*Aporcelaimellus*) and 26.40%, 22.40%, and 19.60% (*Acrobeles*) in CK, T1, and T2, respectively. The proportion of positive correlations in the nematode network gradually decreased from 97.93% (CK) to 92.68% (T1) and 87.97% (T2), while the proportion of negative correlations increased from 2.07% to 7.32% and 12.03%, respectively (**Figures 5a–c**).

**Figure 5:**
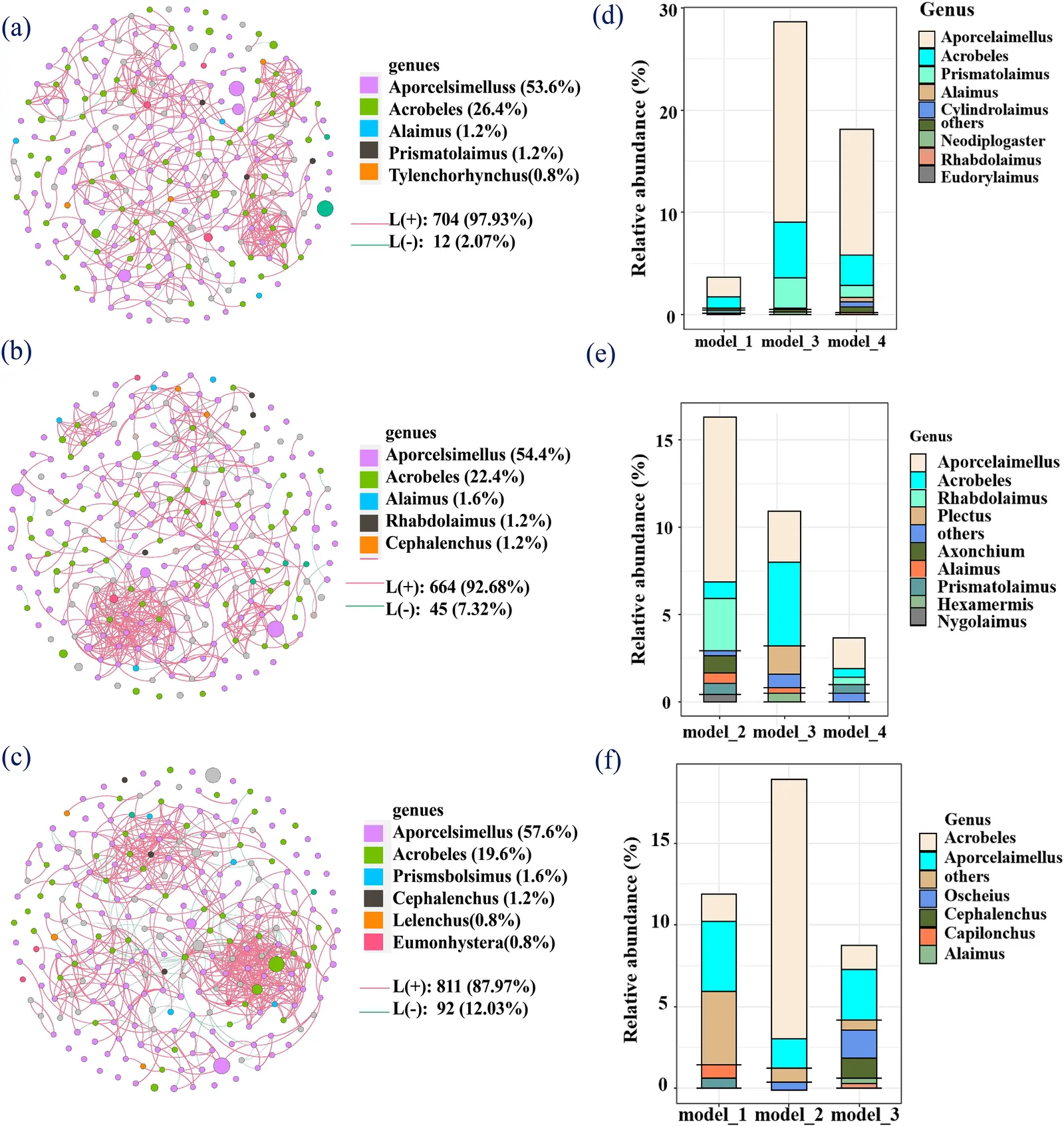
Co-occurrence network analysis of the soil nematode community under different restoration treatments. **(a–c)** Networks of the soil nematode community in the control, mixed seeding, and mixed seeding with Sheep manure treatments, respectively. **(d–f)** Composition of nematode taxa within the main network modules for the Control, Mixed seeding with Sheep manure treatments, respectively.

In CK, both *Aporcelaimellus* (1.94%-19.57%) and *Acrobeles* (1.14%-55.46%) were present in high proportions within the three high-abundance modules (**Figure 5d**). Under T1, *Aporcelaimellus* dominated module 2 (9.43%), while *Acrobeles* dominated module 3 (9.43%; **Figure 5e**). Under T2, *Acrobeles* comprehensively dominated all modules, with its highest relative abundance (15.89%) in module 2 (**Figure 5f**), indicating a shift toward bacterivore-dominated network architecture under manure amendment.

Compared with CK, both T1 and T2 exhibited higher average degree and lower clustering coefficient (**Supplementary Table 2**), suggesting increased network connectivity but reduced local clustering. The overall modular structure remained stable across treatments, indicating that core functional groups (*Aporcelaimellus* and *Acrobeles*) maintained the fundamental food-web skeleton despite species-level reconfiguration.

### 3.5 Correlation analysis between community composition and influencing factors

Redundancy analysis (RDA) and Monte Carlo permutation tests at the amplicon sequence variant (ASV) level revealed that environmental factors collectively explained 11.69% of the variation in nematode community composition at the genus level (**Figure 6**). *Acrobeles* was positively correlated with available potassium (AK), aboveground biomass (AB), and belowground biomass (BB). Monte Carlo permutation tests indicated that available nitrogen (AN) showed the strongest marginal association with genus-level community changes (*p* = 0.065), though it did not reach conventional statistical significance (**Supplementary Table 3**). Other factors, including SOC, EC, and pH, contributed to the remaining explained variation but with weaker individual effects.

**Figure 6:**
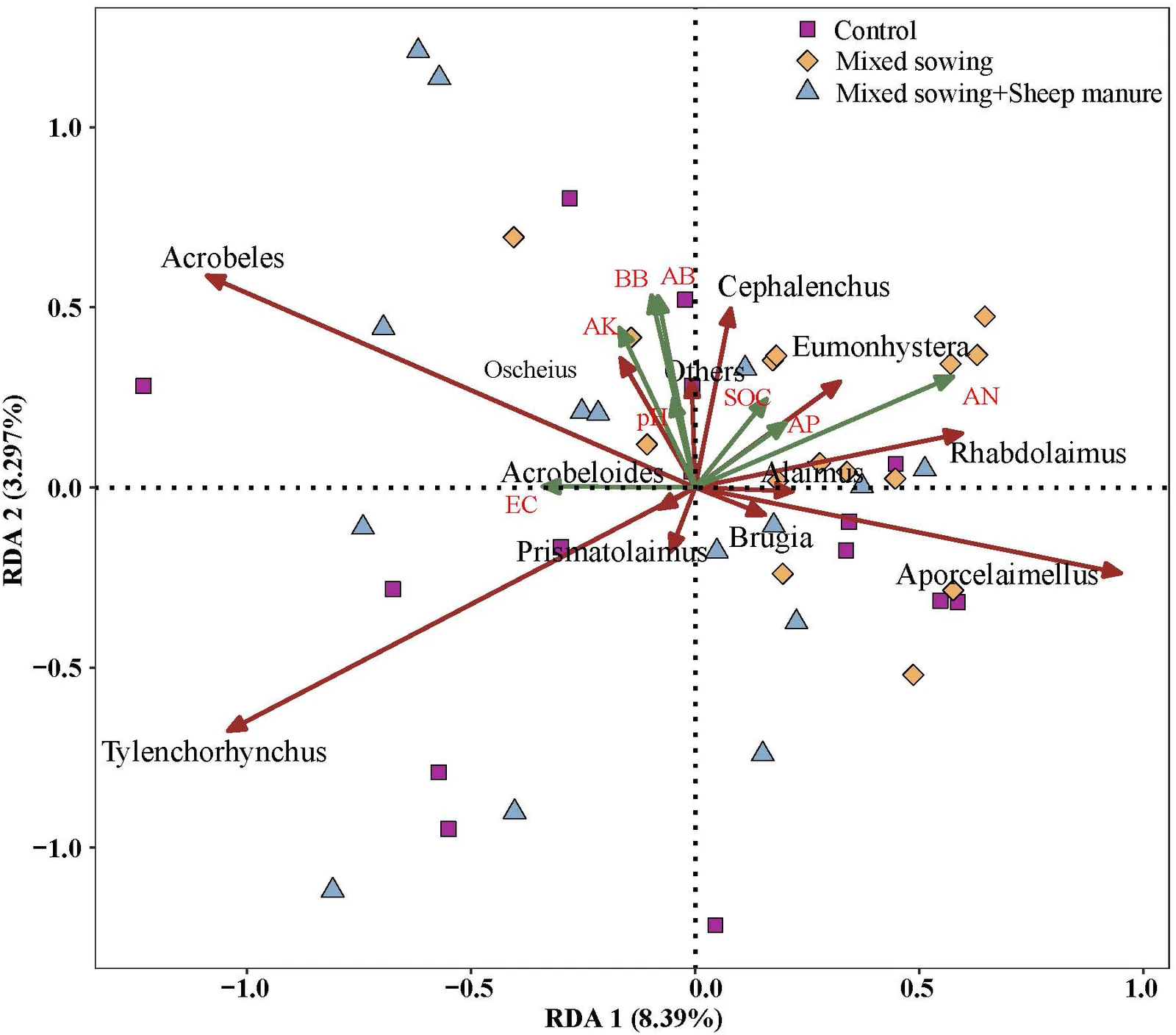
Redundancy analysis of nematodes community and environmental factors. AB, aboveground biomass; BB, belowground biomass; SOC, soil organic carbon; EC, electrical conductivity; AN, available nitrogen; AP, available phosphorus; AK, available potassium.

## 4 Discussion

### 4.1 Effects of restoration measures on environmental factors

For degraded alpine grasslands characterized by soil impoverishment, sparse vegetation, and high sensitivity to environmental disturbances, vegetation establishment and organic fertilizer application have been proven as effective measures to improve soil conditions and promote plant growth (Dai et al., 2020; Bai et al., 2021; Hu et al., 2022; Zhao W. et al., 2024; Zhang et al., 2025). Consistent with our first hypothesis, mixed seeding significantly increased plant biomass in desertified alpine grassland, and this effect was further enhanced when combined with sheep manure amendment, with the mixed seeding plus sheep manure treatment showing the most pronounced effect (**Figure 2**; **Supplementary Table 1**). This improvement can be attributed primarily to the functional complementarity of mixed plant species, (e.g., differences in rooting depth and nutrient acquisition strategies), which enhances resource-use efficiency, and to the direct input of sheep manure, which improves soil fertility and stimulates microbial activity. Together, these mechanisms synergistically strengthen the functioning of the “plant-soil-microorganism” system (Ahmed et al., 2024; Fan et al., 2025; Wang et al., 2025; Wang, 2025).

Consistent with our hypothesis, enhanced plant growth directly altered soil properties. Both treatments significantly increased soil organic carbon, available nitrogen, and electrical conductivity, while decreasing available potassium. In contrast, available phosphorus and pH showed no significant changes (**Figure 2**). In the restoration of desertified alpine grassland, mixed seeding increased SOC primarily through the natural accumulation of root exudates and plant residues, representing a more stable carbon sequestration pathway (Ascenzi et al., 2025). Although the combined application of sheep manure rapidly enhanced productivity, the “priming effect” triggered by the input of exogenous carbon may accelerate the turnover of the native soil carbon pool, complicating the net carbon sequestration effect—a process that requires long-term monitoring (Ai et al., 2012; Bowles et al., 2014). The increase in available nitrogen mainly stemmed from the direct input of high-nitrogen sheep manure (total nitrogen content: 45.04-57.05 g kg^-1^) and the vegetation recovery-promoted nitrogen mineralization process (Zhu et al., 2023; Fan et al., 2026). The significant rise in EC was predominantly caused by soluble base ions introduced with the manure (Legay et al., 2020; Zhu et al., 2021). While this indicates improved soil nutrient availability and confirms the short-term yield-enhancing effect, it also highlights the need to monitor potential salinity balance issues under long-term application. Furthermore, the decrease in available potassium was likely related to the intensified depletion of the soil potassium pool following the substantial increase in plant biomass. Although no significant differences in available phosphorus were observed among treatments, this reflects the complexity of soil phosphorus cycling: new phosphorus sources supplied by manure input and root-mediated activation were largely offset by strong chemical fixation in the soil (Jia et al., 2017; Ali et al., 2023). Similarly, the lack of significant change in pH indicates a dynamic balance between the acidification driven by organic matter decomposition and the combined effects of the soil’s strong buffering capacity and the input of base ions from the organic fertilizer (Wang et al., 2024; Cheng et al., 2025).

In summary, mixed seeding and organic fertilizer application significantly regulate key soil environmental factors in desertified alpine grasslands by altering plant functional combinations and soil resource inputs. These changes inevitably further influence soil biological communities, particularly the diversity, composition, and interaction networks of nematodes. Future research could focus on the long-term monitoring of carbon sequestration stability and how alterations in soil chemical properties shape the structure and function of belowground food webs through cascading effects.

### 4.2 Effects of restoration measures on nematode community diversity and composition

Nematodes, as the most abundant metazoans in soil, are key regulators of soil food-web structure and function (Zhou et al., 2022; Kitagami et al., 2022). Previous studies have shown that excessive use of chemical fertilizers may reduce nematode diversity, whereas organic fertilizer addition can enhance it (Liu et al., 2013; Su et al., 2021; Sun et al., 2025). In this study, we applied mixed seeding with sheep manure amendment, acknowledging that the experimental design does not allow us to isolate the independent effect of manure from its interaction with vegetation reconstruction. We found that, compared with the control, both restoration treatments significantly increased nematode richness but had differentiated effects on community composition. However, the Shannon diversity index of nematodes did not change significantly. Consistent with findings by Sun et al. (2025), organic amendments stimulated the growth of bacterivorous nematodes. Specifically, mixed seeding increased the relative abundance of predators/omnivores, indicating that vegetation reconstruction provided more abundant resources for higher trophic levels (Thakur and Geisen, 2019). This suggests a shift in energy flow within the food web from the fast bacterial channel toward more complex trophic cascades, marking an enhancement in system complexity and internal biological regulation—a key biomarker of ecosystem recovery toward a more mature and structured state (Wang et al., 2022; Xin et al., 2024). However, whether this structural complexity translates into functional stability requires long-term empirical validation. In contrast, the mixed seeding combined with sheep manure treatment reduced the relative abundance of predators/omnivores. This is because the “resource pulse” from exogenous sheep manure preferentially stimulated the explosive growth of fast-reproducing bacterivorous nematodes (r-strategists, such as Rhabditida), thereby proportionally diluting the slower-growing predators (K-strategists, such as Dorylaimida) (Wan et al., 2022; Shi et al., 2023). Contrary to our hypothesis, the restoration measures did not significantly alter the Shannon diversity index of nematodes. Possible reasons include: (1) the exogenous nutrient input only triggered the outbreak of a few opportunistic species without synchronously elevating overall diversity; (2) the reassembly of soil biological communities lagged behind the rapid improvement in abiotic conditions; and (3) harsh environmental factors such as low temperatures in alpine regions imposed strong selective pressures, limiting the responsiveness of diversity to short-term resource inputs. This indicates that during the early restoration stage, the structure of the soil food web exhibits greater resilience than the abiotic environment, and its full response may require a longer time to become apparent (Liu et al., 2025). However, the absence of a sheep-manure-only treatment precludes us from determining whether the observed effects on nematode communities are driven by (1) the direct effect of manure, (2) the interaction between manure and vegetation, or (3) the indirect effect of manure-enhanced plant growth on soil conditions. Future experiments should include a manure-only control to disentangle these mechanisms.

The *Aporcelaimellus* prefers fertile soil (positively correlated with organic carbon, available nitrogen, and available phosphorus) but is sensitive to salinity (negatively correlated with electrical conductivity) (Nguyen et al., 2021). In this study, the mixed seeding combined with sheep manure treatment significantly improved soil nutrients, but also caused a sharp increase in electrical conductivity due to elevated salt ions. It can therefore be inferred that the negative effect of salinity stress may have offset the potential benefits of increased nutrients, explaining why *Aporcelaimellus* did not increase in the treatment with the most abundant resources. This reflects the complex interactive effects of multiple soil factors on biological communities (Cao et al., 2021; Wang et al., 2025). LEfSe analysis revealed that the biomarker in untreated bare sandy soil was the fungivorous nematodes *Ditylenchus*, indicating a simple food web dominated by fungal decomposition pathways in the early stage of degradation (Wang et al., 2018; Zhao F. et al., 2024). The mixed seeding treatment promoted the development of K-strategists such as nematodes in the Enoplea and higher trophic levels, signaling a shift toward a more structurally complex food web (Bongers and Bongers, 1998). This increased trophic complexity represents altered interaction potential, but does not inherently imply enhanced stability without temporal perturbation experiments. In contrast, the addition of sheep manure induced a strong resource disturbance, leading to an outbreak of r-strategists represented by bacterivorous nematodes such as the *Acrobeloides*. Their dominance suppressed community evenness, and together with possible salinity stress, maintained the food web in a relatively simple state in the short term (Liang et al., 2009; Wang et al., 2022; Long et al., 2024).

### 4.3 Reconstruction of interaction network structure of the soil nematodes

Network analysis reveals profound impacts of restoration measures on the organizational patterns of nematode communities at the level of overall interactions. The predatory/omnivorous nematodes *Aporcelaimellus* and the selective bacterivorous nematodes *Acrobeles* served as the network cores across all treatments. This explains why the overall modularity of the network remained stable despite drastic reorganization at the species level—these two core functional groups form the basic skeleton of the food web. However, under both restoration treatments, the centrality of *Aporcelaimellus* within the network consistently increased (its proportion rising from 53.60% to 57.60%), which may indicate that in resource-enriched environments, the relative importance of top-down predatory regulation in network connectivity is enhanced (Pothula et al., 2022). Both measures increased the complexity of the nematode networks but reduced cooperative relationships while increasing competitive interactions (**Figure 5**). This shift toward greater competitive structuring may reflect intensified niche differentiation among nematode taxa under improved soil conditions. While competitive networks can theoretically enhance robustness to perturbation, this one-year cross-sectional design cannot empirically validate such stability claims. This structural reconfiguration suggests increased network complexity (Wang et al., 2017); however, whether this complexity confers greater robustness to perturbation remains to be tested. This shift resulted in a higher average connectivity within the networks, but it also disrupted locally tight symbiotic clusters, leading to a decrease in the clustering coefficient (Liu et al., 2024).

The succession of internal module structure further visually reflects differences in dominant ecological processes. In the mixed seeding treatment, the network modules exhibited functional partitioning, with predators (*Aporcelaimellus*) and bacterivores (*Acrobeles*) dominating modules 2 and 3, respectively. This suggests a clearly structured food web with distinct trophic-level specialization. In contrast, under the mixed seeding combined with sheep manure treatment, the *Acrobeles* comprehensively dominated all modules, indicating that the strong resource pulse induced by exogenous organic fertilizer input shifted the system toward a eutrophic state characterized by convergent energy flow centered on bacterivorous functions (Chen et al., 2014a).

## 5 Conclusion

This one-year artificial grassland restoration experiment demonstrated that mixed seeding and mixed seeding combined with sheep manure amendment significantly affected the richness index and β-diversity of the soil nematode community in desertified alpine grassland, but did not significantly alter the Shannon diversity index. Correlation analysis suggested that available nitrogen showed the strongest marginal association with community composition changes. Network analysis indicated that both restoration measures increased structural network complexity but reduced cooperative relationships within the network, reflecting altered potential interaction patterns rather than verified stability enhancement. The combination of mixed seeding with sheep manure may induce soil salinity stress (EC) and shift nematode communities toward bacterivore-dominated assemblages, thereby altering the structure of the soil nematode community. Due to the absence of a sheep-manure-only treatment, these effects cannot be attributed solely to organic fertilization; they likely reflect the combined or interactive effects of vegetation reconstruction and manure amendment. Therefore, the key to the effectiveness of future ecological restoration lies not merely in the intensity of resource input, but in understanding the specific ecological processes triggered by different restoration configurations. Future studies should incorporate a manure-only control to isolate the independent contributions of vegetation reconstruction and organic amendment.

## Data Availability

All raw sequencing data have been deposited in the NCBI Sequence Read Archive under the BioProject accession number PRJNA1516587.
