# Supplementary material for: Mixed seeding with sheep manure amendment alters soil nematode network complexity in a sandy grassland
Source: Front Plant Sci. 2026 Sep 29;17:1912216. doi: 10.3389/fpls.2026.1912216 (PMC13658175; doi:10.3389/fpls.2026.1912216)
Supplement: Supplementary file 1 [file DataSheet1.docx]

**Supplementary Material**

**Mixed seeding with sheep manure amendment alters soil nematode network complexity in a sandy grassland**

**Table S1** Results of linear mixed-effects models (LMMs) and Tukey’s post-hoc comparisons for plant, soil, and nematode community variables

|  | **NumDF** | **DenDF** | **F_value** | **p_value** | **CK_vs_T1** | **CK_vs_T2** | **T1_vs_T2** |
| --- | --- | --- | --- | --- | --- | --- | --- |
| AB | 2 | 39.00024243 | 126.3058847 | **9.16E-18** | 4.78E-13 | 1.21E-13 | 0.000909629 |
| BB | 2 | 39.00007059 | 115.0879482 | **4.37E-17** | 2.29E-12 | 1.22E-13 | 0.000807218 |
| SOC | 2 | 39.00305453 | 13.73107362 | **3.06E-05** | 2.50E-05 | 0.003542616 | 0.21426036 |
| AN | 2 | 39.08027582 | 72.29529073 | **7.37E-14** | 2.63E-13 | 0.019976268 | 2.57E-10 |
| AP | 2 | 39.00203803 | 0.944765786 | 0.3974868 | 0.999557947 | 0.45379871 | 0.483401523 |
| AK | 2 | 39.13161229 | 10.00797341 | **0.000308828** | 0.303447898 | 0.015025897 | 0.000252168 |
| pH | 2 | 41 | 1.977406423 | 0.151410881 | 0.989848214 | 0.232923377 | 0.194486369 |
| EC | 2 | 38.99920085 | 45.05313772 | **7.28E-11** | 0.000119587 | 3.29E-11 | 9.43E-05 |
| Chao1 | 2 | 38.99934141 | 6.82721153 | **0.002869366** | 0.053099046 | 0.002338479 | 0.491218695 |
| Shannon | 2 | 41 | 0.375729162 | 0.689128561 | 0.711462756 | 0.996476488 | 0.758321974 |

Note: Post-hoc columns show Tukey-adjusted p-values for pairwise comparisons. Significant P-values (P < 0.05) are in bold.

**Table S2** Changes of topological properties of nematodes community network under different restoration measures

|  | Control | Mixed sowing | Mixed sowing with sheep manure |
| --- | --- | --- | --- |
| Number of vertices | 250 | 250 | 250 |
| Number of edges | 531 | 560 | 665 |
| Average degree | 4.25 | 4.48 | 5.32 |
| Diameter | 18 | 25 | 11 |
| Clustering coefficient | 0.59 | 0.53 | 0.51 |

Note: Isolated nodes were retained when calculating the number of vertices and average degree. Edges represent unique undirected associations after collapsing reciprocal records. Average degree was calculated as 2E/N. Diameter was calculated within the largest connected component. The clustering coefficient represents global transitivity.

**Table S3** Monte Carlo test of environmental factors and nematodes community

|  | **RDA1** | **RDA2** | **r^2^** | **Pr(>r)** | **Significance** |
| --- | --- | --- | --- | --- | --- |
| AB | -0.16172 | 0.98684 | 0.0658 | 0.265 |  |
| BB | -0.19455 | 0.98089 | 0.0669 | 0.258 |  |
| SOC | 0.65032 | 0.75966 | 0.022 | 0.633 |  |
| EC | -0.99973 | -0.02322 | 0.0341 | 0.503 |  |
| pH | -0.21502 | 0.97661 | 0.0144 | 0.734 |  |
| AN | 0.91591 | 0.40138 | 0.1228 | 0.065 | ***** |
| AP | 0.81949 | 0.5731 | 0.0201 | 0.631 |  |
| AK | -0.41791 | 0.90849 | 0.052 | 0.335 |  |

“*”: p < 0.10; AB: aboveground biomass; BB: belowground biomass; SOC: soil organic carbon; EC: electrical conductivity; AN: available nitrogen; AP: available phosphorus; AK: available potassium.
